# Sustainable luxury purchase behavior in the Post-Pandemic Era: a grounded theory study in China

**DOI:** 10.3389/fpsyg.2023.1260537

**Published:** 2023-10-16

**Authors:** Huaxia Yang, Xiaoyan Su, Kojo Shion

**Affiliations:** ^1^Land and Tourism College, Luoyang Normal University, Luoyang, China; ^2^Tourism Management College, University of Sanya, Sanya, China; ^3^Faculty of Economics, The International University of Kagoshima, Kagoshima, Japan

**Keywords:** sustainable consumption, Post-Pandemic Era, luxury purchase, grounded theory, purchase behavior

## Abstract

Despite the increasing sustainable practices from the luxury industry, research show little evidence on consumers’ reactions toward sustainable luxury. This paper aims at understanding changes in consumers’ consumption behavior toward sustainable luxury fashion products in the Post-Pandemic Era. We use exploratory qualitative research conducted by semi-structured in-depth interviews involving luxury consumers in China based on grounded theory. With the help of Nvivo12, the interview data is coded and analyzed to identify the influencing attitude and intention of sustainable luxury fashion in China in the Post-Pandemic Era. The findings show that hedonic value, uniqueness value, social value, functional value (practicality, quality, and value for money), health value and sustainable value affect purchase intention toward sustainable luxury in the Post-Pandemic Era. A theoretical model is built to systematically analyze the determinants of purchase behavior. This study will contribute to the literature on sustainable luxury fashion behaviors by performing in-depth interviews in the Post-Pandemic Era. Besides, the findings provide guidance for industries to make customer-based sustainable strategies when practicing sustainable development in luxury fashion in China.

## 1. Introduction

In recent years, global luxury companies adopted environmental corporate social responsibilities (CSR) practices to improve resource use efficiency (Yang et al., 2022) and influence consumer attitudes toward the luxury products (Schill and Godefroit-Winkel, 2022). Previous research on sustainable consumption has focused on examining the effect of sustainable marketing on customer engagement (Kumar Kar and Harichandan, 2022; Gong et al., 2023), the role of CSR in the luxury context (Amatulli et al., 2018; Broccardo et al., 2023) and sustainable supply chain in shaping consumers intentions (Clift et al., 2013; Hu et al., 2019; Li et al., 2022). Factors of sustainable consumption in the luxury context involve intrinstic customer values like hedonic value, functional value and social value (Gazzola et al., 2017; Yang et al., 2022). However, few scholars have explored the impact of the COVID-19 pandemic on sustainable behavior in the context of luxury industry.

The COVID-19 pandemic has led to a decline in fashion consumption (Pang et al., 2022). Researchers have explored the impact of COVID-19 on the fashion industry and consumer behavior by countries including the Finland, the South Korea and Australia (Brydges et al., 2021; Vătămănescu et al., 2021; Pang et al., 2022; Haukkala et al., 2023). Luxury fashion, leading the fashion industry, has adopted new strategies to reduce the influence of the pandemic through the product/monetary donations, e-retailing, the price adjustment strategies and the fashion show strategies in the Post-Pandemic Era (Xie and Youn, 2020). Pang et al. (2022) identified that sales of luxury fashion goods at offline department stores have grown after COVID-pandemic.

Under the influence of the pandemic, there have been many changes in the consumer behavior (Xie and Youn, 2020). Consumers are expected to make decisions more responsibly, taking high quality, durability and adherent to sustainable principles into account when purchasing goods (Vătămănescu et al., 2021). Fashion industry, on the other hand, generates huge waste and pressure on the environment and is allegedly as the second largest industrial polluter in the world (Pelikánová et al., 2021). Studies have revealed the development of sustainable luxury fashion have become of prime importance since the outbreak of the pandemic (Brydges et al., 2021; De Silva et al., 2021). However, although International luxury companies have significantly strengthened their commitment toward sustainable development (Davies et al., 2012; Janssen et al., 2013) and commitment along the supply chain (Gucci group, LVMH) (Cervellon, 2013), sustainable luxury consumption could still be the biggest challenge to many luxury marketers, looking into the long-term economic impacts of the COVID-19 pandemic (Ing et al., 2021). This is partly because the primary role of luxury fashion, unlike other luxury segments where products are made to last (e.g., cars), is to bring innovative authentic designs with premium quality to the world through constant change (Godart and Seong, 2014), and luxury fashion emphasizes the seasonal product development, renowned designer involvement, directly owned stores, flagship experience, and international fashion shows (Fionda and Moore, 2009). However, only a few studies have looked at purchase intention on sustainable luxury consumption (Gazzola et al., 2017; Kapferer and Michaut-Denizeau, 2019; Jain, 2019b) in the Post-Pandemic Era.

The rapidly growing luxury consumption in the emerging economies in Asia, especially China, has helped boost the luxury industry in recent decades. Before COVID-19, China’s luxury market reached 33% of the total revenue in the global luxury consumption (Bain, 2019). It is expected to exceed the United States and become the world’s top market. The pandemic changed the increasing pattern, but the personal luxury goods industry saw the heels of the V-shaped rebound in 2021. Although China continues to confront challenges due to Covid lockdowns and is still performing below 2021 figures, China’s luxury market is expected to recover in a rapid rate in 2023 (Xinhua, 2023). China, along with western fashion brands emerging to locate production lines, is influenced by the negative environmental effects brought from the fashion industry. Consumers also maintain high levels of sustainability awareness, and this is reflected on tangible purchases (Henninger et al., 2017). Nevertheless, excessive consumption is prevalent in China due to the diversity of people, places, perspectives, and preferences (Cervellon and Shammas, 2013). With the growing attention paid to luxury consumption in China (Gao et al., 2009; Li et al., 2012; Zhan and He, 2012; Zhang and Kim, 2013; Songer, 2014; Sun et al., 2016; Hung et al., 2018; Jiang et al., 2019; Kim et al., 2019), and the increasing awareness of sustainable consumption in China (Wang et al., 2014; Dermody et al., 2015; Qu et al., 2015; Liu et al., 2016; Gazzola et al., 2017; Geng et al., 2017), it is critical to explore the drives of sustainable luxury fashion products. In the field of sustainable luxury fashion, although researchers have explored the differences of purchase intention among different countries like the United Kingdom, China, India and the United Arab Emirates (Mishra et al., 2021; Wang et al., 2021; Kaur et al., 2022), there are relatively few studies on the factors influencing consumer sustainable luxury purchase intention in the Post-Pandemic Era (Ing et al., 2021). The only two existing studies are from Ing et al. (2021) and Hála et al. (2022). The former study shows significant roles of experiential, functional, symbolic value perceptions and social influence in forming of luxury purchase intention during the time of pandemic, while the latter indicate there are many differences in customers’ attitudes and habits as well as differences between luxury fashion businesses, their strategies and values. Therefore, the objective of this study is to propose a theoretical model of consumers’ intention toward sustainable luxury fashion goods in China in the Post-Pandemic Era. The results of this study will provide valuable insights to luxury fashion marketers to engage in effective marketing strategies for better survival and stay competitive in China in the Post-Pandemic Era.

The remainder of this study is organized as follows: the first section explores literature written on the concept of sustainable luxury fashion followed by purchase intention and luxury consumption in the post-pandemic ear. The second section explores research methodology which contains data collection and presents data analysis. In the third and last section, after a saturation test, a construction of the theoretical model of consumer behavior toward sustainable luxury fashion is proposed, which provides theoretical and practical implications for this study.

## 2. Literature review

### 2.1. Sustainable luxury fashion

The question of the compatibility and consistency between sustainable development and luxury has puzzled researchers for about a decade. The incompatibility between luxury and sustainability is mainly due to the overconsumption, ostentation and indulgent characteristics of luxury consumption (Veblen, 1899). Researchers point out the irrelevance of luxury and sustainability (Davies et al., 2012; Joy et al., 2012; Henninger et al., 2017), because luxury values highlight personal pleasure and wasteful and careless behavior (Cervellon and Shammas, 2013) while sustainability is linked to altruism, sobriety, moderation and ethics (Janssen et al., 2013; De Angelis et al., 2017). For example, ephemeral luxury products (e.g., clothing) are short-term oriented (De Angelis et al., 2017) compared with enduring luxury products (e.g., jewelry) and thus are perceived as less socially responsible (Janssen et al., 2013). However, other researchers assert that luxury associated with quality, respect for materials, craftsmanship and durability (Vigneron and Johnson, 2004; Kapferer, 2010; Joy et al., 2012) is highly compatible with corporate social responsibility (CSR) (Osburg et al., 2021). They believe luxury products and sustainable development share many characteristics: rarity and beauty (Kapferer, 2010), natural material, slow manufacturing, skilled workforce, local manufacturing, heraldic tradition, vintage value and philanthropy (Cervellon, 2013; De Angelis et al., 2017). Athwal et al. (2019) defined sustainable luxury fashion is that luxury fashion products focusing on pursuing sustainable development, incorporating design, production, and consumption with the goal of balancing the development of the economy, environment, and society. Thus, we need to redefine what sustainable luxury means since the outbreak of the COVID-pandemic.

### 2.2. Purchase intention

Behavior intentions refer to the attitudes or motivations when one intends to take a specific action (Ming-Shen et al., 2007). Several studies within marketing and consumer research have focused on consumer’s purchase intention toward luxury (Summers et al., 2006; Hung et al., 2011; Bian and Forsythe, 2012; Shukla, 2012; Chattalas and Shukla, 2015; Salehzadeh and Pool, 2017; Ku and Lin, 2018; Wang et al., 2018; Zhang and Cude, 2018; Jain, 2019a; Aw et al., 2021). However, only a handful of research focuses on the factors driving consumers’ sustainable fashion and sustainable luxury fashion intention. For instance, Joy et al. (2012) analyzed the perception of consumers in Hong Kong and Canada and found that young consumers separate luxury fashion from sustainability even though they tend to be more ethical. This might be due to consumers’ limited knowledge and awareness of sustainable fashion products (Han et al., 2017). Cervellon and Shammas (2013) pointed out sustainable luxury consumption links with consumers’ socio-cultural values, ego-centered values and eco-centered values in mature market. Ki and Kim (2016) based on self-determination theory, examined consumers’ intrinsic values and extrinsic values and how they influence sustainable luxury purchases. Jain (2019b) discussed the influencing factors of sustainable luxury fashion in an emerging country of India. He proposes a conceptual model based on a literature review by combining the TRA (Ajzen, 1991) with Schwartz’s value theory, but it is lack of empirical test. Later, Kaur et al. (2022) examined the impact of materialism (possessiveness, envy and non-generosity) along with attitude on the purchase intention of sustainable luxury products in India. A recent investigation by Wang et al. (2021) surveyed 677 responses from China and the United Kingdom, suggesting that the need for exclusivity in sustainable luxury items is negatively related to consumers’ purchase intentions in China, while the need for conformity and hedonic needs are positively related. However, most of these studies were conducted in mature markets before the COVID-pandemic. Researchers agree certain contexts affect consumers’ perceptions of sustainable luxury (Rolling and Sadachar, 2018); thus, it is worth exploring whether consumers in emerging markets, like China, perceive the high value of sustainability in the context of the luxury fashion industry in the Post-Pandemic Era.

### 2.3. Sustainable luxury fashion consumption in the post-pandemic era

The COVID-19 pandemic has disrupted fashion activities globally, including fashion weeks canceled, stores closed and workers furloughed, causing industrial crises (Brydges et al., 2021), and the collapse of the global supply chain has increased the production costs of fashion products, including luxury fashion items (Pang et al., 2022) and force luxury fashion industry to adjust their current practices and make changes to navigate their future (Zhao and Kim, 2021). This has also affected the sentiments of Chinese consumers. The volume of luxury items purchased decreased 10%ending a five-year run of exponential growth due to Covid-19-related lockdown (Bain, 2023). The reduction in demand caused by the pandemic has resulted in financial pressure that threatens sustainability initiatives previously (Brydges et al., 2021). In the Post-Pandemic Era, luxury companies that have restarted their operations in China still face multiple problems, such as a labor shortage, shortages of raw materials, and a substantial cost increase in shipping and logistics (D’Arpizio et al., 2020).

Consumers’ habits and priorities for sustainable luxury fashion have also changed (Xu and Nuangjamnong, 2022). Consumers are still afraid of virus-spreading crowds and engage in less face-to-face contact (Fihartini et al., 2021). As lifestyles have changed rapidly to indoor modes, in-store purchases have decreased and the major market channels have switched from offline to online (Xie and Youn, 2020). In 2022, international luxury brands achieved a rapid growth of 31% in online transactions in China in the Post-Pandemic Era, contributing more than half of the market growth (Mak, 2023). Ironically, consumers’ interest in sustainable fashion products, in particular sustainable luxury fashion, has increased since the outbreak of COVID-19 (Vătămănescu et al., 2021). Xu and Nuangjamnong (2022) found there are changes in Second-Hand Clothing-Sharing Consumption Behavior in the Post-Pandemic Era in China.

## 3. Methodology

Research on sustainable luxury consumption of consumers purchase intention in the Post-Pandemic Era is in early stage, which justifies a qualitative inquiry (Henninger et al., 2017). To achieve the purpose of this study, in-depth interviews have been conducted. Analysis of the interviews progressed using the grounded theory approach, which was first put forward by Glaser and Strauss (1967), and constantly revised by scholars, making the theory gradually mature. This study focuses on the purchase intention of sustainable luxury fashion. Grounded theory has significant advantages for theory generation in the field of marketing and consumer behavior (Goulding, 1998; Fischer and Otnes, 2006). It is aimed at building a preliminary conceptual framework of sustainable luxury purchase intention. Therefore, it is appropriate to use grounded theory to study the driving factors of sustainable consumption behavior in sustainable luxury fashion category.

In this study, the coding process was performed with NVivo 12, a computer-assisted qualitative analysis software developed by QSR (Yang, 2022), which is widely used to quantify qualitative data (Liu et al., 2019). The process involves coding strategies (Goulding, 1998): open coding, axial coding, and selective coding. Open coding requires grouping similar incidents together and giving the same conceptual label (Pandit, 1996). All the transcription of interviews were analyzed and coded to identify connections from the investigation (open coding) (Yang, 2022). Axial coding is to identify the internal logical relationship between the initial category and the main category (Cao et al., 2019). Selective coding is the process of identifying core categories (Liu et al., 2019). After passing the theoretical saturation test, the theoretical model of driving factors of sustainable luxury consumption behavior is constructed (Yang, 2022).

### 3.1. Data collection

This study is based on semi-structured interviews, which allows for flexibility and the ability to ask questions outside of the interview guide (Bryman and Bell, 2011). This is carried out by asking questions to understand the perceived sustainable luxury and drivers of purchase intention. Through this process, interview respondents can give direct and deep answers to help better understand motivation and a sense of sustainable luxur y (Yang, 2022). The interviews were conducted both face-to-face and by telephone. The interview time ranged from 19 min to 57 min. This research was conducted from November 19, 2022, to January 15, 2023. Part of the interviewees responded in English although they were all Chinese while most participants chose to speak Mandarin when they were unable to express their opinion sufficiently. All the responses were translated into English. Transcriptions were analyzed line by line qualitatively with NVivo Pro 12 software (Yang, 2022).

To ensure the validity and representativeness of the data, theoretical sampling has been performed (Yang, 2022). Since participants should be able to discuss the luxury purchasing experiences in detail and their perceptions of sustainable development in luxury fashion, only consumers who have purchased luxury fashion products within 2 years were invited to this study (Yang, 2022). A total of 40 usable interviews with Chinese middle and higher class whose household earning is above 160,000 (McKinsey, 2023) were completed per the requirements of theoretical sampling. Thirty-four effective interviews were obtained after the interviews’ assortation, while 6 interviews were excluded due to unsatisfactory quality (Table 1).

**Table 1:** Interviewees’ basic information.

	Characteristics	Number	Percentage
Gender	Female	17	50%
	Male	17	50%
Age	18–20	3	8.8%
	21–25	17	50%
	26–30	5	14.7%
	31–35	7	20.6%
	36–40	2	5.9%
Education	Undergraduate	15	44.1%
	Postgraduate or above	19	55.9%
Lifestyles	Sustainable	7	17.6%
	Potential sustainable	22	67.7%
	Unsustainable	5	14.7%

Results show that the proportion of males and females was half-half (50%). The age 21–25 was the greatest at 50% followed by age 31–35 years old (20.6%) and 26–30 years old (14.7%). All the respondents were undergraduate (44.1%) and postgraduate or above (55.9%), indicating the high level of education of respondents. Apart from the general demographic characteristics, this study, following Qu et al. (2015)‘s methods, added lifestyles to categorize Chinese consumers (sustainable consumers, potential sustainable consumers, and unsustainable consumers) based on their values and attitudes toward sustainable consumption.

### 3.2. Data analysis

#### 3.2.1. Open coding

This study begins with open coding, ‘the process of breaking down the data into distinct units of meaning’ (Goulding, 2000). All the original transcripts were analyzed line by line with the help of Nvivo12 while vague or irrelevant statements were excluded. The factors affecting sustainable luxury consumption were classified and relevant sentences from the interview materials are coded with an unbiased attitude and open mind (Yang, 2022). Three hundred thirty-four items were obtained for note creation, and through browsing coding and extracting relevant concepts, 30 initial categories (label A) have been formed as shown in Table 2.

**Table 2:** Open coding.

Representative quotations from the interviews	Initial concept
If the sustainable luxury products have creative design, for example adding personalized components, I may buy it.	A1: creative choice
I would like to choose the novel/unpopular products that are usually not favorable by many others.	A2: Novelty, avoid of similarity
I like creative accessories or shoes that can perfectly match my identity.	A3: Personal identity
I do not like to follow others’ choices; if other people around me buy an LV handbag, I would definitely not choose the same one even though I like it	A4: Distinctive
I sometimes shop for luxury due to great stress, and I want to forget my work for a short time.	A5: Relax
You know, when I hang around in the luxury brand shop, seeing the creative window display and the warm smile from the employees especially when they are beautiful girls, I feel very stress releasing.	A6: Stress releasing
The luxury fashion I buy, whether sustainable or not, must fit my own taste.	A7: Personal taste
My mom sometimes looks for a beautiful dress for me. When she says, “it really fits you,” I might spend money on that luxury dress.	A8: Family member, friends, or others’ referent
I shop for special occasions because it’s the age to wear something high-end, especially when you go to a party or academic meeting.	A9: Social occasion
Well, to some extent it is a kind of showing off, to let others know that I have an LV handbag.	A10: Face, vanity
My information of luxury fashion is from key opinion influencers, like famous stars on social network. After trying on in retailer shop, if that one fit on me, I would purchase.	A11: Social influencer; Key opinion leaders
I always want to be key low. I would follow the others’ choice.	A12: Conformity
Luxury is a reflection of success or status especially when you attend important meetings.	A13: Status
The sustainable luxury products must have distinctive quality.	A 14: Good quality
Most others would not have the financial ability to purchase.	A15: Premium price
If they choose recycled material, they need to make sure the long-lasting quality. I would consider cost per wear, using luxury for different occasions with traditional black or white colors.	A16: Long-lasting, cost per wear
The (sustainable) luxury fashion I buy must be of authentic design, which can differentiate from ordinary products.	A17: Authentic design, differentiation
Sustainable luxury must be of great service especially after sale services so that consumers would have no concern or doubt about the new high-tech recycled materials	A18: Service
Each Luxury brand has its brand history. The products I buy must be well-known brands and the one I like most.	A19: Brand value
I care about the price. If the price of sustainable luxury is higher only due to sustainability, I definitely would not buy this one.	A20: Relative price, Value-for-money
I think luxury clothes made from natural materials would be comfortable and healthy for us.	A21: Health, natural materials
I believe the new sustainable luxury would do little harm to the environment.	A22: Environmentally friendly Value
The packaging such as boxes should be able to be recycled. Some luxury brands like Mulberry would collect your old products to get recycled.	A23: Recycling
I might consider second hand handbags or accessories instead of clothes.	A24: Second hand
I will definitely not buy any luxury fashion made from wild animal furs. Wild animal furs should not be a trendy, which is terrible for the nature.	A25: Animal-friendly value
Cheap labor is a normal phenomenon, but I hope the employees’ living conditions would be improved when they work for luxury brands.	A26: Cheap labor or sweatshop
If consumers get informed about the pollution from fashion industry by showing the relationship between each purchase and the amount of pollutant, I will change my behavior to buy sustainable luxury fashion.	A27 Sustainable Awareness, knowledge
Luxury industry should set good example at performing sustainable development or socially responsible strategies, so that other fashion industry would follow their trend.	A28: Sustainable product value
If we are told the sustainable story behind the clothes, and at the same time other values are the same, I would definitely buy sustainable ones for their contributions to the environment.	A29: purchase intention
I would like to buy sustainable luxury fashion with a 15% higher price at most.	A30: willingness to pay more

#### 3.2.2. Axial coding

Axial coding is achieved by specifying relations by constant comparison, to group previous codes and to form final theory, the core categories (Goulding, 2005; Valor, 2007). Categories, defined by Corbin and Strauss (1990), are higher in level and more abstract than the concepts they represent (p. 7). This process is the basis of theory construction (Goulding, 2005). The study aims to find out the internal logical relationship between the initial category’s factors that affect consumers’ purchase intention toward sustainable luxury fashion (Yang, 2022). This process incorporates refining the 30 initial categories from open coding and finally forming six core categories after recombining (Table 3).

**Table 3:** The categorization process of influencing factors of sustainable luxury fashion.

Initial category	Sub-category	Core category
A1: Creative choice	Creativity	Uniqueness value
A2: Novelty, avoid of similarity	Avoid of similarity	
A3: personal identity	Self-identity	
A4: Distinctive	Uniqueness	
A5: Relax	Personal hedonic value	Hedonic value
A6: Stress releasing		
A7: personal taste		
A8: Family member, friends, or others’ referent	Social referent	Social value
A9: Social occasion	Social needs	
A10: Face, vanity	Face	
A11: Social influencer; Key opinion leaders	Social influencer	
A12: Conformity	Conformity	
A13: Status	Status	
A14: Good quality	Quality	Functional value
A15: Premium price	Premium price	
A16: Long-lasting, cost per wear	long-lasting	
A17: Authentic design, differentiation	Design	
A18: Service	Service	
A19: Brand value	Brand value	
A20: relative price, value-for-money	Value-for-money	
A21: Health, natural materials	Health	Health value
A22: Environmentally friendly materials	Eco-friendly value	Sustainable value
A23: Recycling		
A24: Second hand		
A25: Animal-friendly value	Animal-friendly value	
A26: Cheap labor or sweatshop	Social responsibly value	
A27 sustainable awareness	Environmental concern	
A28: Sustainable value		
A29: willingness to pay	sustainable intention	Intention
A30: willingness to pay more		

The core categories are uniqueness value, hedonic value, social value, functional value, health value, and sustainable value (Yang, 2022). ‘Uniqueness value refers to consumers’ personal interests or tastes that are differentiated from others when purchasing sustainable luxury fashion. Hedonic value involves consumers’ emotions, such as stress release or relaxation. Social value refers to consumers’ decisions being affected by others’ evaluations or suggestions. Functional value refers to sustainable luxury products’ general qualities and value for money which can satisfy consumers’ requirements. Sustainable value refers to new sustainable qualities including environmental-friendly, social-responsible, or animal-friendly qualities’ (Yang, 2022, p. 132).

#### 3.2.3. Selective coding

In the selective coding process, all the concepts should be pulled together to explain the phenomenon and should have theoretical significance from the data (Goulding, 2005). Through repeated comparison and analysis, it is found that uniqueness value, hedonic value, social value, functional value, and sustainable value are the driving factors of sustainable luxury consumption; egoism and altruism are two aspects that directly influence the sustainable attributes of luxury. By integrating the existing literature, a theoretical model of influencing factors of sustainable luxury purchase intention has been formed (Yang, 2022).

### 3.3. Saturation test

Grounded theory conclusions can only be reached when the data is saturated and sufficient theory has emerged (Cao et al., 2019). The remaining seven interview materials were left to determine whether the theoretical model achieved theoretical saturation by using Nvivo 12 software. If new data or information appears, it would be added to the data pool before the coding process. When the data or information is saturated and enough theory is obtained, the test is passed, and the theory is stable (Aldiabat and Le Navenec, 2018). After the theoretical saturation test, there is no new category other than uniqueness value, hedonic value, social value, functional value, and sustainable value, which indicates the determinants of sustainable luxury purchase have been fully explored. Therefore, it can be concluded that the four factors determine consumers’ luxury purchase intention on sustainable fashion products (Yang, 2022).

## 4. Results and discussion

Based on the analysis above, the factors influencing sustainable luxury fashion consumption are grouped into two main categories: sustainable luxury value and intention. Sustainable luxury value contains the hedonic value, uniqueness value, social value, functional value, health value, and sustainable value. The intention includes consumers’ willingness to pay sustainable luxury and willingness to pay a high price for sustainable luxury (Li et al., 2012).

### 4.1. Hedonic value

According to the results of data analysis, most consumers are egoistic: they buy luxury products only for their own happiness and ignore the effects on the environment (Yang, 2022). ‘Quality, the need for uniqueness, the degree of preference, or hedonism came first; the last thing I consider is eco-friendly even though I am an environmentalist because I pay too much money on luxury goods (I8) (Yang, 2022).

### 4.2. Uniqueness value

All the interviewees express that the luxury fashion products they buy must be different from others and this is seen as a ‘Need for Uniqueness’ (Snyder and Fromkin, 1977; Tian et al., 2001). ‘Sustainable features might increase my satisfaction after purchasing, but what I care about most is whether the product can make me different’ (I9). Consumer’s uniqueness value is ‘an individual’s pursuit of differentness relative to others that is achieved through the acquisition, utilization, and disposition of consumer goods for the purpose of developing and enhancing one’s personal and social identity’ (Tian et al., 2001, p.50). It contains three characteristics, namely ‘avoidance of similarity,’ ‘creative choice,’ and ‘unpopular choice’ (Miremadi et al., 2011). For example, ‘I do not like to follow others’ choices; if other people around me buy (Lascu and Zinkhan, 1999) an LV handbag, I would definitely not choose the same one even though I like it’ (I27). ‘If the sustainable luxury products have a creative design, for example adding personalized components, I may buy it’(I17). ‘I would like to choose the novel/unpopular products that are usually not viewed as favorable by many others’ (I19) (Yang, 2022).

Natural materials may lead to unique designs and individuality, which can match the costs of sustainable fashion with increased perception of value (Lundblad and Davies, 2016). Therefore, the luxury marketers should personalize the luxury fashion products and incorporate the brands’ corporate social responsibility initiatives by using sustainable materials to match one’s personal style (Kim et al., 2016).

### 4.3. Social value

Social value affects sustainable luxury purchase intention (Jain, 2019b). Many researchers have explored how social value plays an important role in shaping consumers’ purchase intention especially when products are seen as luxury goods (Amaldoss and Jain, 2008). Face culture has a great influence on Chinese consumers’ consumption values due to the social prestige need which can generate a bandwagon effect (Jap, 2010). Similarly, this study further supported this view (Yang, 2022). ‘I think we buy luxury items not because of the products themselves, but due to the Me-tooism. Friends around me always buy new luxury fashion…shoes for example; I think if I get the limited edition, I will show off. Face comes first when I make luxury decisions; then followed by the need to get recognized by others’ (I5) (Yang, 2022). Another factor is seen as social identity, which is defined as ‘a perception of oneness with a group of persons’ and ‘social identification stems from the categorization of individuals, the distinctiveness and prestige of the group’(Ashforth and Mael, 1989) (p20). ‘Clothes make the man. If a man wears luxury clothes, it may feel different’ (I21). ‘I was addicted to luxury products for a short time in the past. At that time, I believed that would be a package for me and I wanna be that kind of person. I sometimes search for key words like ‘how to be an exquisite girl’ and influenced by others recommendations about luxury branded fashion products’ (I3) (Yang, 2022).

Activating status motives led people to choose green products, especially when shopping in public (Griskevicius et al., 2010). Young consumers’ green purchasing intentions are positively influenced by social and emotional values (Awuni and Du, 2016). ‘If a green consumer buys sustainable products including luxury ones, it may increase the social identity around the green friend circle’ (I8) (Yang, 2022).

### 4.4. Functional value

#### 4.4.1. Practicality

Although some consumers buy luxury products for collection as I20 mentioned: ‘I may buy limited edition just for collection’, most interviewees purchased luxury fashion products for personal use, although the price is much higher than the cost (Yang, 2022). Post-80s interviewees express their stronger utilitarian motivation than their post-90s counterparts; this is mainly because they have job experience, they are more mature, and are less impulsive when purchasing (Yang, 2022).

‘The luxury fashion product I bought must be useful. I am a foreign student in the United Kingdom, but my family is not as rich as many others. I purchased a luxury branded bag because I can use it for a long time’ (I18). ‘I only buy luxury products when I really need them. I believe in the high quality of a luxury brand, but I will not buy it for fun or for face’ (I23) (Yang, 2022).

#### 4.4.2. Quality

Superior quality of luxury goods is an important motive for consumers to purchase, which is strongly linked to the product attribute long-lasting (Lundblad and Davies, 2016). Interviewees view price as a cue of quality assessment, and they take it for granted. ‘I believe luxury shoes or clothes own the best quality compared with other branded products’ (I19). Consumers’ expectation toward luxury products is unconsciously high in terms of quality; this is seen as perceived quality, which refers to ‘the consumer’s judgment about a product’s overall excellence or superiority’ (Zeithaml, 1988, p. 3). ‘Many luxury products are handmade or have a sophisticated design, and this is part of their core competitiveness in terms of superior quality’ (I24) (Yang, 2022).

On the other hand, interviewees also mentioned ‘the quality depends on brands’ (I26) or ‘similar premium quality might also be found in not luxury fashion products’ (I22). If luxury products use recycled material, they might doubt the quality (I27). Thus, in common with Cervellon (2013), the industry should improve the technology to ensure good quality and sustainability simultaneously, instead of altering the authenticity of luxury brands (Yang, 2022).

#### 4.4.3. Value for money

In contrast to prior research suggesting that price affects consumers’ purchase decisions less significantly compared with other factors (Jain, 2019b), and consumers perceiving value in noneconomic terms for making sustainable consumption decisions (Lundblad and Davies, 2016), this study shows for sustainable luxury goods, consumers do care about price. Price is one of the major determinants of sustained green consumption behavior (Yang, 2022); the intention to pay the green price premium is the outcome of sustained green consumption (Biswas and Roy, 2015). ‘The product price in western countries is much lower than that in China’ (I5). ‘I would buy more luxury products when I travel in foreign countries because the luxury products are cheaper than the same ones in mainland of China’ (I8) (Yang, 2022).

This study found that consumers perceive that sustainable products need consumers to pay more. ‘I believe sustainable consumption to some extent equals luxury consumption because satisfying this need costs, resulting in high prices (I7). However, some consumers would not buy very high-priced sustainable luxury products (Yang, 2022). ‘If the fashion product increased price only due to the sustainable strategy, I would not decide to buy it immediately but get more information about this product. If the whole manufacturing process including most of the materials changed, I would like to think about it; otherwise definitely not’ (I27). ‘Eco-friendly products are usually expensive; if the design does not fit me and the price is high, I would not buy it. Most students would choose relatively low-priced luxury products’ (I17) (Yang, 2022).

### 4.5. Health value

Consumers purchased sustainable luxury fashion products because they are self-benefit, and well-being can be incorporated with sustainability in this respect (Henninger et al., 2017). Respondents show strong purchase intention for healthy products, and they would like to pay more money for healthy goods especially in the Post-Pandemic Era. ‘Health is very important. I have heard about a piece of news about a bracelet which is made from poisonous chemical materials; if a child unintentionally eats a bead from it, the child may die’ (I4). ‘I think clothes made from natural material would be comfortable and healthy to use’ (I20). Similarly, consumers would like to pay more for healthy products if they know the effect. ‘I would like to pay twice the price on luxury cosmetics only due to the natural and healthy original materials’ (I10) (Yang, 2022).

### 4.6. Sustainable value

Consumers’ sustainable value is linked to their lifestyles. Respondents in this study hold significantly different attitudes toward sustainable luxury products as they have various lifestyles in terms of sustainable development (Yang, 2022). This study adopted Qu et al. (2015)‘s classification method according to consumers’ values and attitude toward sustainable consumption, which divided consumers into three groups: sustainable (Group 1), potential sustainable (Group 2) and unsustainable consumers (Group 3). Group 1 consumers are those who highly care about the environment, and protecting the environment is part of their lifestyle; therefore, they would like to engage in sustainable consumption, hoping to help reach a balance between human beings and nature (Yang, 2022). They are against unsustainable behavior and would like to actively take part in sustainable consumption. ‘When I use a tissue, I would tear it up into three pieces to use. I would turn off the light when not needed. I would try my best to save energy’(I8) (Yang, 2022). These consumers would like to buy sustainable alternatives although the price would be higher than normal luxury products.

Most respondents are in the group 2 spectrum while a few are highly sustainable consumers (Table 4). Group 2 sees sustainable consumption as positive behavior to protect the environment and they believe people should conduct sustainable consumption, but they are very sensitive to the price of products and only would like to insist green purchasing when the government provided rewards (Qu et al., 2015). Additionally, green marketing would to some extent impact their consumption behavior if some external incentives are provided. According to the survey, the majority of the respondents are belonging to Group 2, which means that Chinese consumers (Yang, 2022).

**Table 4:** Consumers segmentation.

Group	Naming	Feature and behavior	Response to sustainable marketing	Interviewees (I)
Group 1	Sustainable consumers	Commit to protect the environment; conduct sustainable consumption as part of lifestyle	Yes	8, 11, 20,22,25,29,32
Group 2	Potential sustainable consumers	Sensitive to price; adjust their consumption style according to policies or laws	Yes, but with conditions.	1,2,3,4,6,7,9, 10,12,13,15,16, 17,18,19,23,27,28,30,31,33,34
Group 3	Unsustainable consumers	Unsustainable behavior; Sensitive to price; Not caring about the result of consumption behavior; Only conduct sustainable behavior when it is mandated by the government.	No	5, 14, 21, 24, 26

Source: Author’s analysis from interview responses and adapted from Qu et al. (2015).

Even though some luxury consumers are well educated and know the importance of sustainable development, there are still a few consumers who are unsustainable consumers (I5, I14, I21). Group 3 is not aware of what sustainable consumption behavior is and what sustainable consumption is about (Yang, 2022, p. 128). They are sensitive to product prices, indicating if green product prices are more expensive than that of the corresponding products, they would not buy them. Meanwhile, they are not concerned about the product quality or the product packaging. They seldom use reusable substances, and they do not care about the result of their consumption behavior. They also would not want to understand the government policies on sustainable consumption. ‘Marketing campaign would have no effect on my purchase behavior; the impression is just like it’s a big company and that is what the brand should do, but I will buy my own way’ (I21) (Yang, 2022).

### 4.7. Willingness to pay

Sustainable luxury consumers are willing to buy sustainable luxury products with 10–20% higher prices rather than normal luxury goods. ‘The way that fast fashion industry produce is wasteful and excessive consumption is harmful for the environment (I20). What should be noted is their choices depend on the product categories. For instance, for secondhand luxury products, most respondents would not buy secondhand clothes as they mentioned they are ‘neat freaks’ ‘Secondhand handbags are acceptable as some collections are hard to find in luxury retailers (I34)’. By contrast, self-directed people are independent and not likely to be influenced by social norms. The priority of motivation comes from their preference instead of sustainable values.

By contrast, a luxury brand’s sustainable initiatives may also attract consumers, especially those who identify themselves as sustainable consumers, as sustainable luxury fashion allow them to better express their self-identity (Osburg et al., 2021). As for the majority of potential sustainable consumers, they might not represent sustainable choice as their priority, although studies show ethical and sustainable criteria are important to luxury consumers (Janssen et al., 2013; Osburg et al., 2021).

## 5. Implications and future research

The research on the sustainable luxury consumption is at the primary stage, and there is a lack of analysis on what factors influences consumers purchase intention toward sustainable luxury fashion in the Post-Pandemic Era. This study answers Osburg et al. (2021)‘s call for the need to explore consumers’ attitude toward sustainable fashion luxury category.

Firstly, this study enriches the literature on sustainable consumption behavior in the luxury context. Consumers’ behavior is explained by the core categories of sustainable luxury values (hedonic value, social value, functional value, health value and sustainable value) and intention. Previous studies show sustainable luxury consumption contribute to social environmental well-being through brand authenticity, self-appraisal, reflected appraisal, and self-esteem (Pai et al., 2022) and the need to explore how consumers perceive sustainable luxury and what affect sustainable consumption behavior (Sun et al., 2021). The purchase intention of sustainable luxury fashion is identified based on grounded theory to systematically analyze dynamic consumer behavior in the Post-Pandemic Era. The findings highlight the interdependent relationships between diverse values and intention with the aim of capturing the complexities of consumers’ purchase intentions in the Post-Pandemic Era.

Secondly, this study is among the earliest pandemic related sustainable consumer studies in China. The existing pandemic related luxury literature are either during the pandemic (Kumar, 2023) or about supply chain management (Karaosman et al., 2023) and changes to online channels (Klöckner et al., 2022) or case study in mature like Korea (Pang et al., 2022) and Czech (Hála et al., 2022). The findings of the present study indicated that COVID-19 pandemic influence consumers’ luxury fashion purchase intention in China and lead consumers rethink more about sustainability in purchasing behavior. The results indicated that consumers could base on different values in determining their luxury purchase intention in the Post-Pandemic Era.

Third, the research has practical values for luxury industries. Environmental CSR issues present a key strategy for a luxury company’s sustainable development (Broccardo et al., 2023). This study provides a reference for the luxury fashion industry to develop a scientific, local-based, systematic sustainable guidance strategy to better communicate with consumers. Specifically, the target of sustainable luxury should be sustainable consumers and potential sustainable consumers as marketing strategies have little effect on unsustainable consumers. Sustainable consumers would definitely choose sustainable luxury products if they are informed the existence of sustainable luxury bags or clothes that they are looking for. With regards to the majority of potential sustainable consumers who are price sensitive, managers in the luxury industry should provide rewards or incentives like green gifts to inform these consumers when launching communication campaign. As Luxury brand image positively affect sustainable marketing activities (Park et al., 2010), and regular consumers of sustainable fashion seek long term benefits of switching to sustainable luxury brands (Lundblad and Davies, 2016), there is an emergency need for the luxury industry to manufacture sustainable fashion by using terms such as eco-fashion or fair trade fashion in brand communication (Lundblad and Davies, 2016). and upgrade their products’ certification levels and setting sustainable goals as priorities (Shao, 2019).

Marketing campaigns should focus on these product values (unique style, timeless cuts, health benefit of natural material, long-lasting garment) of sustainable luxury fashion in order to increase the number of consumers. The price suggested is no higher than 20% compared with traditional luxury fashion. Consumers’ are ego-centered especially when they pay more money on luxury fashion products compared with low-priced counterparts.

Additionally, through understanding consumers’ changing intentions in the new era, this study provides insight for industrial enterprises to take sustainable actions on supply chain, and to guide consumers to buy sustainable luxury fashion. Luxury brands can leverage sustainability into their marketing strategies instead of just focusing on product excellence in the Post-Pandemic Era (Hála et al., 2022).

This study can serve as the research foundation for further analyzing the sustainable behavior of luxury consumers in the Post-Pandemic Era. Future studies can take quantitative analysis to test the relationships. A questionnaire can be designed by incorporating the identified influencing factors with previous literature to verify the theoretical framework, to examine individual differences based on their demographic characteristics. Cross-country research from other markets could be done to obtain a wider perspective of consumers’ sustainable luxury behavior. This study used purposive sampling method, which has its own limitations; future studies may use probability sampling technique to obtain better generalizability of data.

## Data availability statement

The original contributions presented in the study are included in the article/supplementary material, further inquiries can be directed to the corresponding authors.

## Ethics statement

Ethical review and approval was not required for the study on human participants in accordance with the local legislation and institutional requirements. Written informed consent from the participants was not required to participate in this study in accordance with the national legislation and the institutional requirements.

## Author contributions

HY: Conceptualization, Data curation, Investigation, Methodology, Writing – original draft. XS: Funding acquisition, Investigation, Writing – review & editing. KS: Conceptualization, Data curation, Investigation, Methodology, Writing – review & editing.
